# A Bibliometric Analysis of Disaster Nursing and Management Research: Global Trends, Key Insights, and Future Directions (1980–2024)

**DOI:** 10.3390/nursrep16070236

**Published:** 2026-07-08

**Authors:** Banu Terzi, Fatma Azizoğlu, Duygu Sönmez Düzkaya

**Affiliations:** 1 Department of Fundamentals of Nursing, Faculty of Nursing, Akdeniz University, Antalya 07070, Turkey; 2 Department of Nursing, Faculty of Health Sciences, Haliç University, Istanbul 34060, Turkey; fatmaazizoglu@halic.edu.tr; 3 Department of Nursing, Faculty of Health Sciences, Tarsus University, Mersin 33400, Turkey; duygusduzkaya@tarsus.edu.tr

**Keywords:** bibliometric analysis, disasters, disaster planning, disaster preparedness, emergency nursing, nursing education, Web of Science

## Abstract

**Background/Objectives**: As the frequency and impact of global disasters intensify, the role of nursing in disaster management has become increasingly vital. Analyzing the evolution of disaster nursing research can inform preparedness strategies and enhance clinical responses. The aim of the study was to explore and visualize trends in research on disaster, disaster management, disaster nursing, and disaster preparedness from 1980 to 2024. **Methods**: A scientific mapping and performance analysis were conducted using Biblioshiny, VOSviewer, and Excel. The search strategy included the keywords ‘disaster’, ‘disaster management’, ‘disaster nursing’, ‘disaster preparedness’, ‘disaster training’, and ‘disaster care,’ searched across all fields in the Web of Science Core Collection database. A total of 1331 publications from a dataset of 21,703 articles were included in the analysis. **Results**: The most common keywords were “disaster”, “nursing”, “COVID-19”, and “emergency preparedness”. The USA led in research output, followed by Sichuan University as the top institution. Thearticle, “Disaster preparedness among nurses: a systematic review of literature”, was the most cited, with 216 citations. English was the predominant language, and most publications were articles. **Conclusions**: Research in disaster nursing, management, and preparedness has grown significantly, particularly since 2005, emphasizing the critical need for improved disaster education and response strategies. This study was not registered.

## 1. Introduction

Disasters can be defined as natural, technological or human-induced events that cause serious physical, social and economic losses by interrupting the normal functioning of societies in a sudden and unexpected way, exceeding the coping capacity of the population [1,2,3]. These events have negative impacts on health, economy and environment and may affect a wide area, requiring local, national or international response [4,5]. The impact of disasters encompasses not only emergency relief needs but also the reconstruction and recovery processes of affected communities [6]. The preparedness of communities against disasters and the effective functioning of health systems are critical factors that reduce mortality and morbidity rates while improving survival rates. In this context, disaster management, increasing social resilience and developing rapid response strategies for disasters are of great importance [2].

It is an undisputed fact that healthcare professionals play a critical role in all stages of the disaster management cycle. In particular, the functions of nurses in the post-disaster response process have been proven by various examples around the world and have an important place in the effectiveness of this process [3,5,7]. The active participation of nurses in disaster response directly contributes to reducing the loss of life on both individual and societal scales and alleviating the suffering and losses of the population affected by the disaster [7]. Nurses not only provide basic medical care, but also fulfill important functions such as psychosocial support and health education, accelerating the recovery processes of communities and increasing post-disaster resilience [8,9].

Disaster nursing is a discipline that refers to nurses’ systematic use of disaster-specific knowledge and skills to engage in comprehensive activities to improve health services and public health before, during and after disasters [10]. The roles of nurses in this field go beyond individual patient care and include developing strategies to increase resilience against the social and environmental impacts of disasters, ensuring continuity of health services, and accelerating post-disaster recovery processes [11,12]. Disaster nursing is recognized as a dynamic field of practice that is gaining more and more importance both nationally and internationally [10,13].

The scope of disaster nursing goes far beyond traditional nursing practices. The responsibilities of nurses in disaster situations are not only limited to clinical care provided in hospital settings, but also include health and safety services by adopting a multidisciplinary approach under special conditions such as resource limitations, high levels of psychological stress and emergency management in the disaster area [11,14]. Nurses are involved in a wide range of tasks from post-disaster triage practices to emergency care, psychosocial support and rehabilitation for casualties [12,13].

Disaster nursing also includes activities at the strategic level such as making health services suitable for disasters, reducing disaster risks, and making health infrastructure resilient to disasters [11]. These processes require not only clinical skills but also a range of different competencies such as disaster management, leadership, ethical decision-making, and social resilience [10,15]. The success of disaster nursing requires nurses to be trained in a way that will enable them to assume an effective role in disaster management systems and to develop organizational capacity to be prepared for disasters. In this context, disaster nursing includes knowledge, skills and strategic understandings that will enable nurses to respond flexibly and effectively to extraordinary situations under disaster conditions, and the development of competencies in this field is becoming one of the most critical components of modern health systems [16,17].

Beyond natural and technological disasters, armed conflicts represent a distinct and increasingly prevalent form of disaster that places extraordinary demands on nursing practice. Nurses working in conflict zones face extreme psychological, emotional, and clinical burdens, including trauma exposure, resource scarcity, and deteriorating working conditions. Recent evidence from the ongoing conflict in Gaza highlights the profound impact of war on healthcare workers’ quality of life, with studies documenting severely compromised physical, psychological, and environmental wellbeing among nurses and other health professionals [18,19]. Furthermore, the displacement of populations during armed conflicts introduces additional challenges for nursing practice, particularly in relation to cross-cultural communication and the provision of culturally sensitive care, as demonstrated in studies examining healthcare delivery to Ukrainian war refugees [20]. These findings underscore the critical importance of preparing nurses not only for natural disasters but also for the complex, multifaceted demands of conflict-related humanitarian crises, further strengthening the rationale for comprehensive research in disaster nursing.

Although the importance of nurses in disaster situations is widely emphasized [8,10,12], it is seen that this field is not sufficiently supported, and training in disaster nursing remains inadequate in nursing practice [21,22]. Studies have shown that nurses are not sufficiently prepared for disasters [21,22], they are not equipped with sufficient knowledge and skills [8,14], and this deficiency negatively affects the effectiveness of health services [14,23]. Especially in developing countries, it is reported that the competencies of nurses in disaster management are quite insufficient [12,24]. Therefore, disaster management education programs need to be strengthened and integrated into the nursing curriculum [20,25].

### Aim of Study

Although bibliometric studies on related topics exist in the literature—most notably Molassiotis et al. [26], who analyzed disaster nursing research over a 30-year period (1990–2019)—a comprehensive bibliometric mapping that integrates disaster, disaster management, disaster nursing, and disaster preparedness over a broader time frame (1980–2024) has not yet been conducted. Moreover, previous analyses have not employed the combination of Biblioshiny (Bibliometric analyses were performed using the bibliometrix package in R version 4.3.1-R Foundation for Statistical Computing, Vienna, Austria) and VOSviewer (VOSviewer version 1.6.20, developed by Nees Jan van Eck and Ludo Waltman at the Centre for Science and Technology Studies, Leiden University, the Netherlands) tools simultaneously, nor have they included thematic mapping alongside thematic evolution analysis.

The literature review conducted reveals that no comprehensive bibliometric mapping study has been identified that addresses the concepts of disasters, disaster management, disaster nursing, and disaster preparedness in an integrated manner and covers the period from 1980 to 2024. Accordingly, the present study aims to contribute to the literature in four key ways: (i) by covering a more up-to-date and broader time span (1980–2024) compared to previous studies; (ii) by addressing the concepts of disaster, disaster management, disaster nursing, and disaster preparedness within an integrated framework rather than in isolation; (iii) systematically revealing the conceptual transformation of the literature over time through thematic evolution analysis; and (iv) identifying emerging research areas in disaster nursing that have not yet matured. While the complementary use of the methodological tools (Biblioshiny and VOSviewer) constitutes a methodological choice that enables this comprehensive analysis, the study’s fundamental originality stems not from the use of multiple methodological tools but from the breadth of scope and content outlined above.

Research questions:

- What are the characteristics of publications?
- What are the most prolific authors, countries, institutions and journals?
- What is the network map according to the frequency of use of keywords?
- What is a thematic evolution map?
- What is thematic map analysis?

## 2. Materials and Methods

### 2.1. Research Design

This study employed a bibliometric research design to systematically analyze the intellectual structure, publication trends, and thematic evolution of the disaster nursing and disaster management literature indexed in the Web of Science Core Collection between 1980 and 2024. Bibliometric analysis is a quantitative, non-experimental research design that uses mathematical and statistical methods to evaluate scientific publications and map the structure of a research field [27]. This design is particularly suited to the aims of the present study, as it enables a macro-level examination of research output without the need for primary data collection or human subject involvement. Bibliometric analysis was selected over alternative evidence synthesis approaches, such as systematic reviews or scoping reviews, which synthesize findings on specific clinical questions, because it allows for the identification of influential authors, institutions, and journals, the detection of emerging research trends and gaps, and the mapping of thematic evolution across an entire field over time. These objectives cannot be adequately addressed through traditional evidence synthesis methods [27].

Given the rapid growth of disaster nursing research and the absence of a comprehensive bibliometric mapping study integrating disaster, disaster management, disaster nursing, and disaster preparedness within a single analytical framework covering the period from 1980 to 2024, the present study represents a unique contribution to the field.

### 2.2. Search Strategy and Data Source

The study data were collected from the Web of Science (WoS) Core Collection database. WoS was selected as the sole database because it provides comprehensive coverage of high-impact, peer-reviewed journals across the natural sciences, social sciences, and emerging sources, and is widely recognized as a reliable source for bibliometric analyses [27]. The following search string was used in the WoS database to obtain bibliometric data: “Disaster (Title) AND Disaster management (All Fields) OR disaster nursing (All Fields) OR disaster preparedness (All Fields) AND Disaster Training (Topic) AND Disaster Care (Topic) and 2024 or 2023 or 2022 or 2021 or 2020 or 2019 or 2018 or 2017 or 2016 or 2009 or 2008 or 2007 or 2006 or 2005 or 2004 or 2003 or 2002 or 2001 or 2000 or 1993 or 1999 or 1998 or 1997 or 1996 or 1995 or 1994 or 2015 or 2014 or 2013 or 2012 or 2011 or 2010 or 1980 or 1981 or 1982 or 1983 or 1984 or 1985 or 1986 or 1987 or 1988 or 1989 or 1990 or 1991 or 1992 (Publication Years) and Article or Review Article (Document Types) and Article or Review Article (Document Types) and Nursing (Web of Science Categories) and English or Turkish or Spanish or Korean or Portuguese or German or Italian or Polish (Languages)”. The search was conducted on 24 December 2024, and retrieved a total of 21,703 records. Following the application of inclusion and exclusion criteria, 1331 publications were included in the final analysis.

Inclusion criteria: (1) peer-reviewed research and review articles published between 1980 and 2024; (2) publications indexed in the Science Citation Index Expanded (SCIE), Social Sciences Citation Index (SSCI), or Emerging Sources Citation Index (ESCI); and (3) publications in English, Turkish, Spanish, Portuguese, Korean, German, Italian, or Polish.

Exclusion criteria: (1) conference papers, editorials, letters, book chapters, and early access articles; (2) publications not directly related to disaster nursing, disaster management, or disaster preparedness; and (3) duplicate records. The complete search and selection process is illustrated in Supplementary Figure S1.

### 2.3. Eligibility Criteria and Data Collection

Data were retrieved from the WoS Core Collection database on 24 December 2024. The publications selected for analysis included peer-reviewed articles and reviews, published in journals indexed in the Science Citation Index Expanded, Social Sciences Citation Index, and Emerging Sources Citation Index. However, following the application of inclusion criteria, the final dataset consisted exclusively of articles (*n* = 1331). The selected articles were exported in BibTeX (bibliometrix package in R version 4.3.1, Foundation for Statistical Computing, Vienna, Austria) and Plain Text formats for further analysis. The VOSviewer (version 1.6.20) software and Biblioshiny (bibliometrix package in R version 4.3.1, R Foundation for Statistical Computing, Vienna, Austria) in the R (version 4.3.1, Foundation for Statistical Computing, Vienna, Austria) programming environment were used for performance analysis, scientific mapping, and thematic mapping.

### 2.4. Ethical Considerations

Ethical approval for the study was not required as the research was based on publicly available data from the WoS database, which does not involve direct interaction with human subjects. The study complied with all applicable guidelines for secondary data analysis.

### 2.5. Data Analysis

Bibliometric analysis was performed using the R programming environment, VOSviewer, and Excel. Descriptive bibliometric analysis included examining publication trends over time, author productivity, the most frequently used keywords, and the geographical distribution of publications. In VOSviewer, the minimum occurrence threshold for keyword co-occurrence analysis was set to 5; association strength was used as the clustering method, and lin-log normalization was used as the normalization method. In Biblioshiny, the minimum cluster frequency was set to 4, the number of words to 200, and the number of levels per cluster to 2 for thematic mapping. Thematic maps were generated to visualize the evolution of research topics. Thematic evolution analysis was performed to identify the main themes, motor themes, niche themes, and emerging themes in disaster nursing research. The reliability and validity of the analysis were ensured by using recognized bibliometric tools and by verifying results through different software programs [28,29]. The time periods in the thematic evolution figure were adjusted manually to reflect the actual data retrieval period (1980–2024), as the software automatically generated an extended label beyond the dataset’s scope.

## 3. Results

The findings of the bibliometric analysis are analyzed under five subheadings.

### 3.1. Characteristics of Publications

The analysis identified a total of 1331 publications classified as articles, with an annual growth rate of 1.53%. On average, 6.82 articles were published per year, and each article received a mean of 12.11 citations. Across all publications, a total of 34,137 references were cited, and 2556 author keywords were identified. In addition, 3846 authors contributed to the publications, of which only 184 articles were single-authored. The average number of co-authors per article was 3.66, while the rate of international co-authorship was 14.8%. Dataset-related information was generated using Biblioshiny, and the details are presented in Table 1. English was the dominant publication language (*n* = 1317), followed by Turkish (*n* = 4), Spanish (*n* = 3), Korean (*n* = 2), Portuguese (*n* = 2), German (*n* = 2), Italian (*n* = 1), and Polish (*n* = 1).

Analysis of the annual distribution of publications revealed that the highest number of articles was published in 2021 (*n* = 160, 12.02%). Publication output began to increase notably after 2005, while the earliest publications in the field were identified in 1980 (*n* = 2, 0.15%).

### 3.2. Most Productive Author, Country, Institution and Journal Analysis

The most prolific author was identified as “Veenema TG” (*n* = 52), while the United States ranked first among countries in terms of publication output (*n* = 584). Sichuan University was the leading contributing institution (*n* = 46). Among funding bodies, the United States Department of Health and Human Services provided the highest level of support (*n* = 34). Public Health Nursing was the journal with the greatest number of publications in the field (*n* = 61).

The most highly cited publication was the article entitled “Disaster preparedness among nurses: A systematic review of literature” by Labrague et al. [3], published in International Nursing Review. This study received a total of 216 citations, with an average of 30.86 citations per year.

According to the H-index analysis, which reflects both scientific productivity and scholarly impact, Arbon P had the highest H-index (H-index = 11), followed by Usher K (H-index = 10), Ranse J (H-index = 9), and Veenema TG (H-index = 7). Among journals with high H-index values, the Australasian Emergency Nursing Journal (H-index = 17) and the Journal of Clinical Nursing (H-index = 16) were identified as the most influential sources.

### 3.3. Network Map by Frequency of Use of Keywords

In bibliometric analysis, the frequency and distribution of keywords are used to identify research trends, areas of interest, and patterns of knowledge dissemination within a specific field. A cluster refers to a group of studies organized around a particular topic or research area. Clustering enables the identification of subfields and thematic structures within the literature, as well as the relationships among these themes. A link represents the connection between one publication and another, which may be established through citation relationships, collaborations, or co-authorship patterns. In bibliometric studies, links play a crucial role in understanding the dynamics of scholarly communication and scientific collaboration. Total link strength (TLS) refers to the cumulative strength of a publication’s or author’s connections with other publications. A high TLS value indicates both strong academic influence and dense interconnections within the scientific literature [27,30].

**Figure 1:**
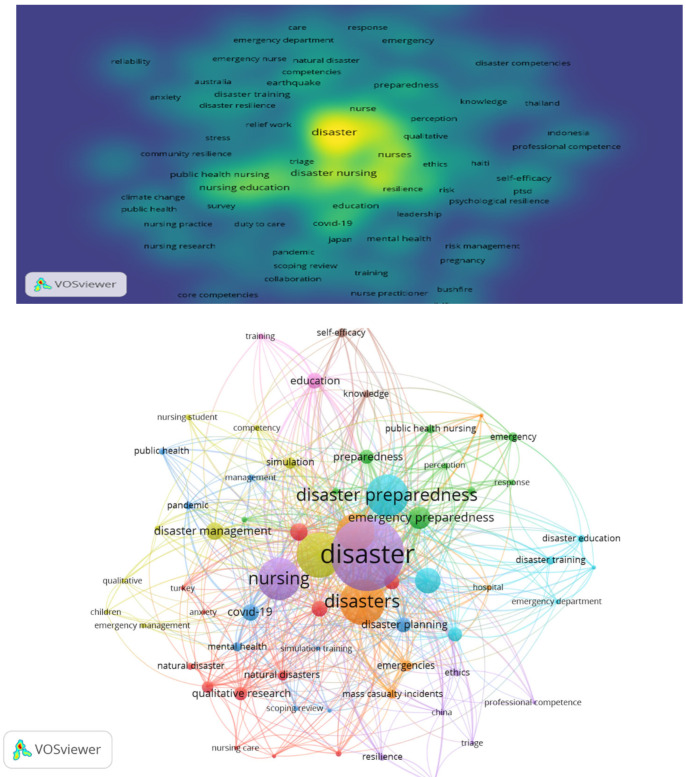
Co-occurrence network and density map of author keywords in disaster nursing and disaster management research (1980–2024). Note: In VOSviewer, the minimum occurrence threshold for keyword co-occurrence analysis was set to 5; association strength was used as the clustering method, and lin-log normalization was used as the normalization method. In Biblioshiny, the minimum cluster frequency was set to 4, the number of words to 200, and the number of levels per cluster to 2 for thematic mapping. The network contains 9 clusters, 65 items, 575 links, and 1216 total link strength (TLS) values. Node size reflects keyword frequency; line thickness reflects co-occurrence strength; colors represent distinct thematic clusters.

In the keyword co-occurrence analysis, a network structure comprising 970 keywords was identified, of which 65 met the minimum occurrence threshold. The resulting network consisted of 9 clusters, 575 links, and a total link strength (TLS) of 1216. Among the most prominent keywords, “disaster” appeared in the 5th cluster with 56 links and a TLS of 222, while “disaster nursing” was located in the 4th cluster with 48 links and a TLS of 132. The keyword “nursing” was also positioned in the 5th cluster with 48 links and a TLS of 152. “Disaster preparedness” was identified in the 6th cluster with 41 links and a TLS of 127, and “emergency preparedness” appeared in the 2nd cluster with 28 links and a TLS of 68. The distribution of these keywords across distinct thematic clusters reflects the multidimensional nature of disaster nursing research and highlights the central conceptual domains that have shaped the literature in this field (Figure 1).

### 3.4. Thematic Evolution Map

**Figure 2:**
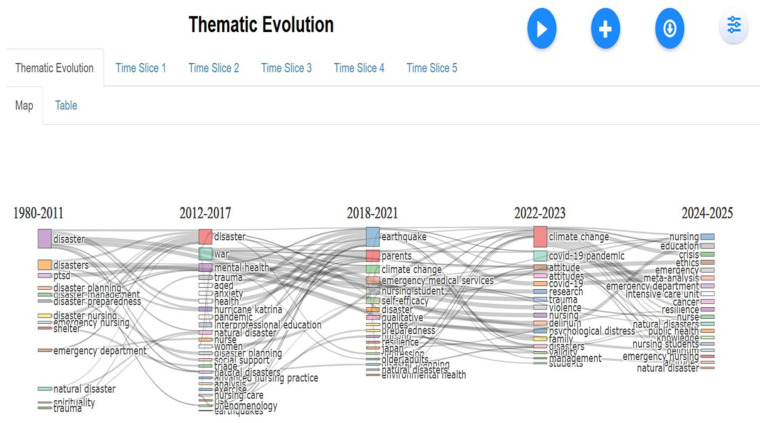
Thematic evolution of publications according to author keywords. Note: Thematic evolution of author keywords across three time periods (1980–2011; 2012–2021; 2022–2025). Generated using Biblioshiny (R programming environment).

The thematic evolution of the disaster nursing and disaster management literature demonstrates how prominent research topics have changed over time. Between 1980 and 2011, foundational themes such as “disaster”, “disaster planning”, and “disaster nursing” were the most dominant topics. During the 2012–2017 period, themes related to major crises and emergency events, including “Hurricane Katrina”, “earthquake”, and “war”, became increasingly prominent. More recently, between 2022 and 2024, themes such as “climate change”, “nursing”, “education”, and “public health” have gained substantial attention and now occupy a central position in the literature (Figure 2).

### 3.5. Thematic Map Analysis

**Figure 3:**
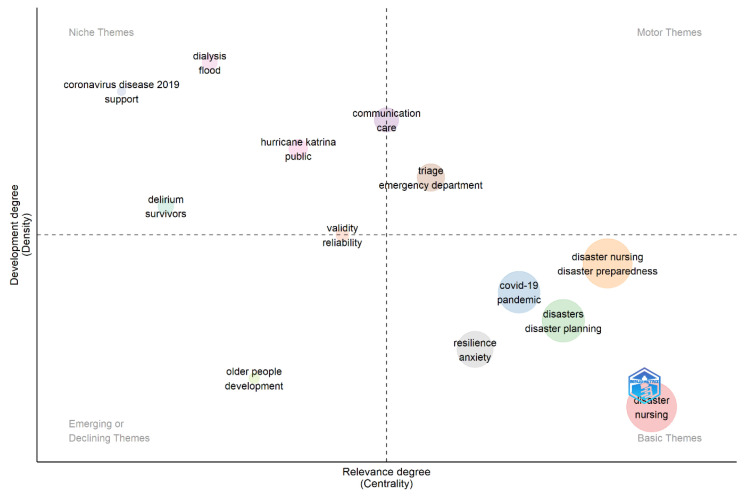
Thematic mapping (Note: Thematic map based on 200 words, minimum cluster frequency = 4, two levels per cluster. Upper right = motor themes; upper left = niche themes; lower right = basic themes; lower left = emerging/declining themes. Generated using Biblioshiny-R programming environment).

The thematic map analysis illustrates the clustering patterns of keywords within the disaster nursing and disaster management literature and highlights the relationships among thematic areas. In thematic mapping, the upper right quadrant represents motor themes, which are characterized by high centrality and high density, indicating well-developed and influential themes within the field. The lower right quadrant includes basic and transversal themes, which have strong connections with other themes but relatively lower internal development. The upper left quadrant contains niche themes, characterized by high density but low centrality, suggesting specialized yet isolated research areas with limited external connections. Finally, the lower left quadrant represents emerging or declining themes, which are characterized by both low density and low centrality [31,32]. The thematic typology of research related to disasters, disaster management, disaster preparedness, and disaster nursing is presented in Figure 3.

In the thematic map analysis, 200 keywords were included, the minimum cluster frequency was set at four, and two levels were defined for each cluster (Figure 3). The motor themes located in the upper right quadrant demonstrated both high density and high centrality and included clusters related to “communication”, “care”, “triage”, and “emergency department”. The niche themes in the upper left quadrant were represented by the clusters “dialysis”, “flood”, “coronavirus disease 2019”, “support”, “delirium”, “survivors”, “Hurricane Katrina”, and “public”. The lower right quadrant, representing basic and transversal themes, included the clusters “disaster nursing”, “COVID-19”, “pandemic”, “disaster”, “disaster planning”, “resilience”, and “anxiety”. The lower left quadrant, which reflects emerging or declining themes, contained keywords related to “older people” and “development”. The thematic map analysis illustrates the clustering patterns of keywords within the disaster nursing and disaster management literature and highlights the relationships among thematic areas. In thematic mapping, the upper right quadrant represents motor themes, which are characterized by high centrality and high density, indicating well-developed and influential themes within the field. The lower right quadrant includes basic and transversal themes, which have strong connections with other themes but relatively lower internal development. The upper left quadrant contains niche themes, characterized by high density but low centrality, suggesting specialized yet relatively isolated research areas with limited external connections. Finally, the lower left quadrant represents emerging or declining themes, which are characterized by both low density and low centrality [31,32]. The thematic typology of research related to disasters, disaster management, disaster preparedness, and disaster nursing is presented in Figure 3.

In the thematic map analysis, 200 keywords were included, the minimum cluster frequency was set at four, and two levels were defined for each cluster (Figure 3). The motor themes located in the upper right quadrant demonstrated both high density and high centrality and included clusters related to “communication”, “care”, “triage”, and “emergency department”. The niche themes in the upper left quadrant were represented by the clusters “dialysis”, “flood”, “coronavirus disease 2019”, “support”, “delirium”, “survivors”, “Hurricane Katrina”, and “public”. The lower right quadrant, representing basic and transversal themes, included the clusters “disaster nursing”, “COVID-19”, “pandemic”, “disaster”, “disaster planning”, “resilience”, and “anxiety”. The lower left quadrant, which reflects emerging or declining themes, contained keywords related to “older people”, “development”, and “reliability” (Figure 3).

## 4. Discussion

Despite the development in disaster nursing and increasing research and related publications, little effort has been made to map the global development and trends of disaster nursing, disaster management and disaster preparedness literature, identify gaps and guide future research in the field. This study provides a comprehensive picture of WoS-indexed publications in the field of disaster nursing over the last 44 years, acknowledging that findings are limited to the Web of Science Core Collection and may not fully represent the global literature.

### 4.1. Evolution and Research Hotspots

The observed increase in publications starting from 2005, with a significant peak in 2021, reflects the growing scholarly attention to disaster preparedness and nursing in response to global events. The notable rise in research output beginning in 2005 may be associated with the expanding recognition of disaster nursing as an important area of healthcare research, potentially coinciding with major disaster events such as the 2004 Indian Ocean tsunami and Hurricane Katrina in 2005, which prompted significant policy and academic responses globally [33,34]. The sharp rise in publications in 2021 is consistent with findings from Yang et al. [35], who documented a significant increase in nursing research output during the COVID-19 pandemic, suggesting that the global healthcare crisis may have substantially accelerated scholarly interest in disaster nursing and preparedness frameworks. These bibliometric trends indicate that the pandemic period was associated with heightened research activity in this field; however, the extent to which this increased output has translated into improved preparedness practices falls beyond the scope of bibliometric analysis.

This growth in publications has been documented by various scholars, such as Labrague et al. [3], who highlighted the rise in research on disaster nursing and preparedness in the wake of large-scale emergencies. Similarly, the work of Yang et al. [35] emphasizes how the COVID-19 pandemic was associated with an increased focus on disaster nursing and preparedness. As global health emergencies continue to rise, bibliometric trends suggest that research in this field is likely to continue growing, with emerging areas such as climate change and pandemics appearing increasingly in the literature.

The keyword network and density map shown in Figure 1 reveal the thematic priorities of the literature on disaster management and nursing. In the network structure, keywords such as “disaster”, “disaster nursing”, “nursing”, and “disaster preparedness” stand out with high density and link strength, indicating that these concepts occupy a central position in the literature and represent a common focus area among researchers. In particular, concepts such as “disaster preparedness” and “emergency preparedness” appear as prominent thematic trends, suggesting that the role of nursing practices in disasters has received sustained scholarly attention [23]. The nine different clusters identified in the map reflect the interdisciplinary nature and thematic diversity of the research. These clusters represent concentrations around specific research areas. For example, the cluster associated with “disaster nursing” reflects growing academic interest in the role of nurses in disasters, while the cluster linked to “emergency preparedness” indicates a thematic emphasis on preparedness and education strategies in the literature [33].

Keyword density and network links can also serve as indicators of relative research concentration, thereby pointing to areas that appear underrepresented in the current literature. Keywords or clusters with low link counts may suggest topics that are less studied or still developing. For example, nursing care in disaster situations and care of older adults in disasters appear to have received comparatively less scholarly attention, as evidenced by their low link counts and peripheral positioning in the keyword network. These patterns suggest that these areas may warrant further research attention; the most appropriate methodological approaches, however, should be determined based on the specific research questions to be addressed [27].

Figure 2 illustrates the thematic evolution of author keywords over time. In the period 1980–2011, foundational themes such as “disaster” and “natural disaster” were predominant. In the period 2012–2017, themes focusing on specific events and strategies—such as “earthquake”, “disaster planning”, and “war”—became more prominent. In the most recent period (2022–2024), themes such as “climate change”, “education”, and “public health” gained visibility, suggesting that research priorities in the literature have shifted toward environmental factors, public health considerations, and educational dimensions of disaster preparedness. This thematic evolution indicates that the literature has increasingly moved toward proactive and anticipatory research agendas; whether this shift has corresponded to meaningful changes in nursing practice or policy, however, cannot be determined from bibliometric data alone [34].

In a similar bibliometric analysis by Molassiotis et al. [26], the main themes of publications include disaster, nurses/emergency nurses/military nurses, preparedness, communication, and information. In disaster nursing, the disaster response phase received the most attention, followed by the preparedness phase, and very few publications addressed disaster mitigation and recovery.

Compared with the findings of Molassiotis et al. [26], who reported a steady but modest growth in disaster nursing publications between 1990 and 2019, the present study identifies a markedly accelerated growth trajectory, particularly after 2005 and peaking in 2021. This acceleration appears to coincide with the impact of the COVID-19 pandemic and the increasing presence of disaster preparedness topics in nursing and health policy literature globally [14,23]; however, causal attribution cannot be established from publication trend data alone. Furthermore, the dominance of the United States in research output, also observed by Molassiotis et al. [26], persists in the present dataset, suggesting that geographical disparities in disaster nursing research remain a structural pattern in the literature. The relatively low international co-authorship rate (14.8%) identified in this study further underscores the limited global collaboration in the field, a pattern consistent with broader trends in nursing research [8,12]. These observed patterns may suggest a need for international research initiatives and funding mechanisms to address existing geographical imbalances, though the effectiveness of such mechanisms falls outside the scope of the present analysis.

### 4.2. Thematic Mapping and Interpretation

1. Motor themes (top right quadrant): *Motor* themes represent the most frequently studied and centrally positioned topics in the literature. These themes include key concepts such as “communication”, “care”, and “emergency department”. The prominence of these themes in disaster nursing literature suggests that communication, patient care coordination, and emergency department management have been consistent focal points in the field [14,22]. The concentration of these themes in the motor quadrant also indicates that they are well-established areas of scholarly inquiry. These bibliometric patterns are consistent with findings from previous studies, which have similarly identified inter-professional communication and triage as frequently discussed topics in disaster response literature [14,22]. Based on these observed trends, simulation-based training in communication and triage may represent a promising direction for nursing education; however, the effectiveness of such approaches should be evaluated through primary research rather than inferred from publication patterns alone [34].

2. Niche themes (upper left quadrant): Niche themes are characterized by low centrality but high density, typically representing narrowly scoped but intensively studied topics. Themes such as “dialysis”, “flood”, and “hurricane Katrina” tend to focus on specific events or contexts. For example, the theme “hurricane Katrina” is associated with studies examining the impact of this natural disaster on healthcare systems and nursing [36]. While these themes provide in-depth knowledge within their respective areas, their limited connections to other themes suggest relatively isolated research trajectories. The concentration of niche themes around specific disaster events such as Hurricane Katrina and COVID-19 may reflect a broader tendency in the literature toward reactive, event-driven scholarly inquiry [36]. These patterns suggest that more anticipatory research agendas addressing emerging disaster risks—such as climate-related disasters and armed conflicts—may be underrepresented in the current literature [11,17].

3. Basic themes (lower right quadrant): Basic themes are defined by high centrality but relatively low density. Concepts such as “disaster nursing”, “resilience”, and “disaster planning” are broadly referenced across the literature but appear to have been studied with comparatively lower depth and specificity [11,17]. The classification of these concepts as basic themes indicates that, while they are widely connected to other topics, the literature may not yet reflect a sufficiently developed or nuanced body of knowledge in these areas. These bibliometric patterns may suggest that more focused research on resilience-building in disaster contexts, as well as the conceptual development of disaster nursing competencies, could represent productive directions for future inquiry [10,15].

4. Emerging or declining themes (lower left quadrant): Themes in the lower left quadrant are characterized by low centrality and low density, suggesting that they either represent newly emerging areas or topics that have received declining attention in the literature. Themes such as “older people”, “development”, and “reliability” appear among the less-studied areas in the current dataset [36]. The peripheral positioning of themes related to older adults in the keyword network may indicate that nursing research specifically addressing this population in disaster contexts remains limited. These patterns highlight older adults and other vulnerable populations as potentially underrepresented areas in the disaster nursing literature; future primary research exploring the specific needs and risks of these groups during disasters may be particularly warranted [12,24].

## 5. Study Limitations

To the best of the authors’ knowledge, this study represents the first comprehensive scientific mapping of research in disaster, disaster nursing, disaster management, and disaster preparedness using a combined Biblioshiny and VOSviewer analytical framework. However, several limitations should be considered when interpreting the results.

First, the data were exclusively sourced from the WoS Core Collection database, which may introduce selection bias by excluding publications indexed in other major databases such as Scopus, PubMed, CINAHL, and Embase, as well as regional databases that may contain important contributions from non-English-speaking countries. Consequently, conclusions regarding the global landscape of disaster nursing research should be interpreted with caution, as the findings reflect the intellectual structure of WoS-indexed literature rather than the entirety of published work in this field. Future studies could broaden the scope by incorporating additional databases to provide a more comprehensive picture.

Second, the search strategy, while comprehensive, may have omitted studies that use alternative or region-specific terminology for disaster-related concepts. International variations or inconsistencies in terminology, such as differing terms used for the same concept across disaster-related disciplines, may have resulted in an incomplete retrieval of relevant publications.

Third, citation counts in bibliometric analyses are inherently subject to temporal bias, as older publications have had more time to accumulate citations compared to more recent work. Similarly, English-language publications and journals indexed in WoS tend to receive disproportionately higher citation counts, which may underrepresent the contributions of non-English-language scholarship and publications indexed in other databases.

Fourth, the present study focuses on disaster nursing and management broadly, without distinguishing between specific disaster subtypes such as natural disasters, technological disasters, and armed conflicts. Given the distinct nursing challenges associated with conflict-related humanitarian crises, future bibliometric analyses should consider disaggregating the literature by disaster type to provide more nuanced insights into each subfield.

Fifth, the term frequency method used in thematic analysis may not be entirely robust, as topic selection across different articles may not be independent. Future research could utilize more sophisticated thematic analysis methods to examine topic trends in greater depth.

## 6. Implications for Nursing Practice

The bibliometric analysis revealed that the international co-authorship rate was 14.8%, which is notably low given the global nature of disaster events. This finding directly suggests the need to foster international research partnerships. Policies that incentivize collaborative research and cross-border knowledge exchange can enhance disaster preparedness strategies on a global scale [8,12,26].

The keyword network analysis identified ‘disaster preparedness’ and ‘resilience’ as basic themes, concepts with high centrality but relatively low research density, indicating that while these topics are widely referenced, they remain insufficiently developed in the literature [10,15]. This bibliometric pattern directly supports the need for nursing education programs to incorporate comprehensive disaster management modules, ensuring that nurses are equipped with the skills and knowledge to respond effectively to disasters, including those arising from armed conflicts and humanitarian crises [18,19,20].

The thematic map analysis identified ‘older people’ and ‘development’ in the emerging/declining themes quadrant, characterized by low centrality and low density, indicating that research targeting vulnerable populations remains significantly underrepresented in the disaster nursing literature [18,36]. This finding underscores the importance of community-centered disaster planning, and policymakers should prioritize education and resources for at-risk groups, including older adults, refugees, and displaced populations affected by armed conflicts, to improve community resilience [18,20].

The thematic evolution analysis revealed that ‘climate change’ and ‘public health’ emerged as prominent themes in the most recent time period (2022–2024), signalling a shift in research priorities toward environmental and public health dimensions of disaster nursing. Evidence from conflict zones further demonstrates that mental health consequences of disasters, whether natural or conflict-related, represent a critical but underaddressed dimension of nursing practice [19,20]. National disaster frameworks must therefore integrate both climate-related risks and mental health considerations to address the increasingly multifaceted nature of modern crises [14,23].

The analysis identified the United States Department of Health and Human Services as the leading funding agency (*n* = 34), while the majority of research output was concentrated in the United States (*n* = 584). This bibliometric evidence of geographical and funding concentration highlights the need for expanding financial support to under-represented regions and under-researched areas such as mental health and long-term disaster recovery, which will strengthen the global evidence base in disaster nursing [12,24,26].

## 7. Conclusions

This bibliometric analysis provides a comprehensive overview of research trends in disaster management, disaster nursing, and disaster preparedness within the Web of Science (WoS) Core Collection between 1980 and 2024, using a combined Biblioshiny and VOSviewer analytical framework. The findings demonstrate a substantial increase in publication productivity over time, particularly following major global crises such as the COVID-19 pandemic, while also identifying influential authors, institutions, journals, and thematic research clusters within WoS-indexed literature.

The analysis revealed that the intellectual structure of the field has evolved from traditional disaster planning and emergency preparedness toward broader and more contemporary concerns, including climate change, public health resilience, mental health, and vulnerable populations. In addition, the emergence of armed conflict and humanitarian crises as increasingly visible themes highlights the growing complexity of disaster nursing research and the need for more context-specific investigations. However, these findings should be interpreted cautiously, as the analysis was limited to WoS-indexed publications and may not fully represent the entirety of global disaster nursing scholarship, particularly research published in non-English languages, regional journals, or alternative databases.

Despite these limitations, the study offers valuable insight into the development, thematic evolution, and collaborative structure of disaster nursing research. The relatively low level of international collaboration identified in the analysis underscores the need for stronger cross-national and interdisciplinary partnerships, especially in regions that remain underrepresented in the literature. Future bibliometric studies should integrate multiple databases, employ more advanced thematic analysis methods, and examine disaster subtypes separately to provide a more comprehensive and globally representative understanding of disaster nursing research and its contribution to healthcare system resilience.

## Figures and Tables

**Table 1:** Information regarding the dataset.

Description	Results
Timespan	1980:2024
Sources (Journals, Books, etc.)	162
Documents	1331
Annual Growth Rate %	1.53
Document Average Age	6.82
Average citations per doc	12.11
References	34,137
**Document Contents**	
Keywords Plus (ID)	1362
Author’s Keywords (DE)	2556
**Authors**	
Authors	3846
Authors of single-authored docs	167
**Authors Collaboration**	
Single-authored docs	184
Co-Authors per Doc	3.66
International co-authorships %	14.8
**Document Types**	
Article	1219
Review	107
Review; early access	5

## Data Availability

The original contributions presented in this study are included in the article/Supplementary Material. Further inquiries can be directed to the corresponding author.

## Supplementary Materials

The following supporting information can be downloaded at: https://www.mdpi.com/article/10.3390/nursrep16070236/s1; Supplementary Figure S1. Flow diagram.

## Public Involvement Statement

There was no public involvement in any aspect of this research.

## Guidelines and Standards Statement

This manuscript was drafted against the BIBLIO-Checklist.
