# Supplementary figures and images for: A Bibliometric Analysis of Disaster Nursing and Management Research: Global Trends, Key Insights, and Future Directions (1980–2024)
Source: Nurs Rep. 2026 Jul 8;16(7):236. doi: 10.3390/nursrep16070236 (PMC13414519; doi:10.3390/nursrep16070236)

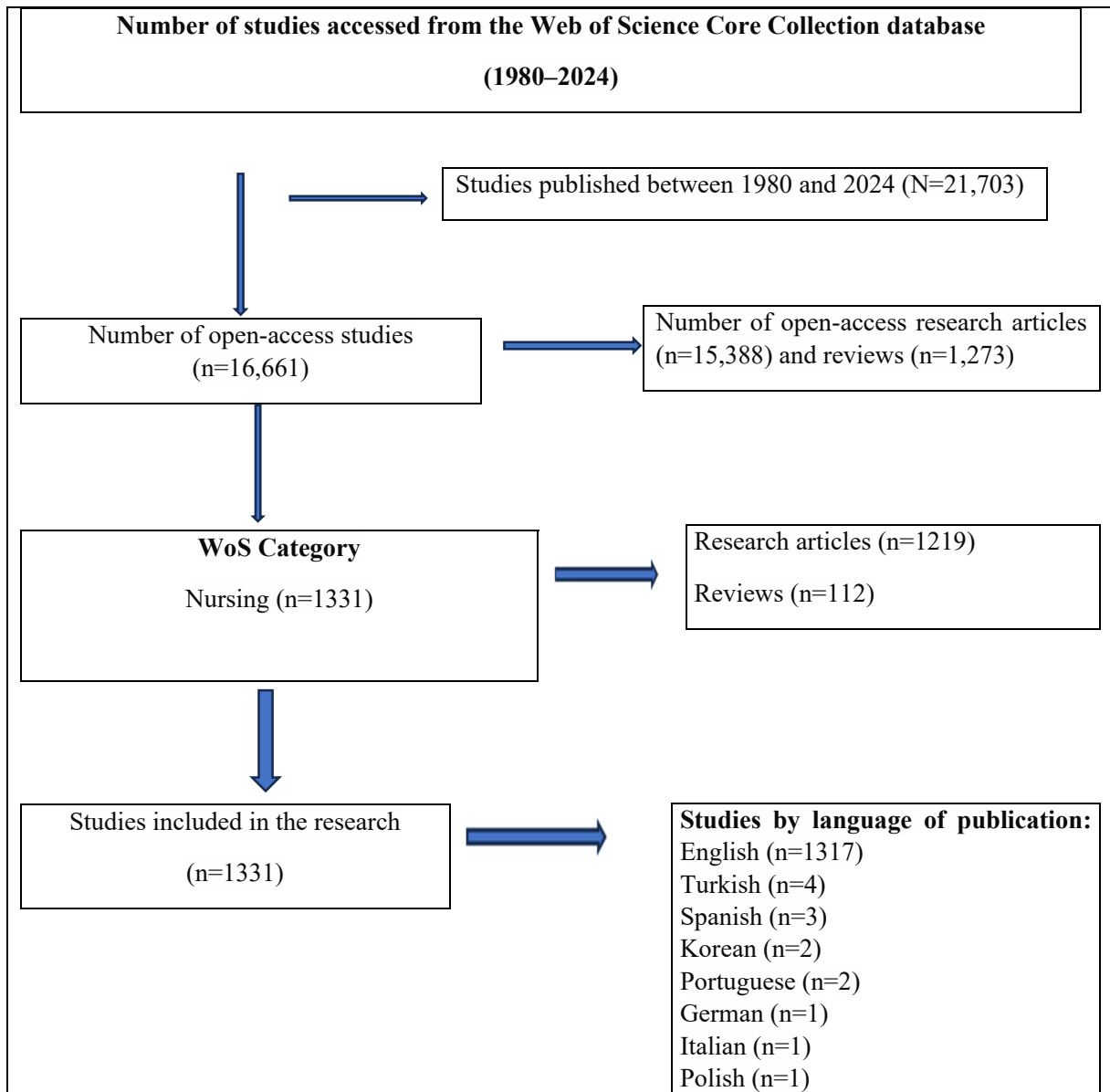

**Supplementary Figure S1.** Flow diagram

Supplement: Supplementary file 1 [file nursrep-16-00236-s001.zip › nursrep-4368370-supplementary.pdf]
